# Support-seeking and rehoming pathways differ by surrender circumstances among pet owners

**DOI:** 10.1371/journal.pone.0345326

**Published:** 2026-03-30

**Authors:** Lexis H. Ly, Shelby E. McDonald, Isain Zapata, Alexandra Protopopova

**Affiliations:** 1 Animal Welfare Program, Faculty of Land and Food Systems, University of British Columbia, Vancouver, British Columbia, Canada; 2 School of Social Work, College of Health and Human Sciences, Colorado State University, Fort Collins, Colorado, United States of America; 3 Department of Biomedical Sciences, Rocky Vista University, Englewood, Colorado, United States of America; UKM: Universiti Kebangsaan Malaysia, MALAYSIA

## Abstract

Animal shelters aim to divert intake by encouraging pet retention in homes (e.g., support services) or through alternative methods of surrender (e.g., self-rehoming); however, it remains unclear what factors contribute to an owner’s decision to seek pet support services, as well as to select different methods to surrender a pet. Using a sample of U.S. and Canadian public members who rehomed a pet within the past five years (n = 452), the present study identified groups of pet owners who share similar patterns of responses to surrender circumstances using latent class analysis (LCA). LCA revealed three heterogeneous classes of owners distinguished largely by the reason for surrender and the length of ownership (Owner Hardships n = 215, New Acquisitions n = 194, Behavioural Incompatibility n = 43). Comparisons revealed differences across classes regarding the proportion of respondents that sought assistance and the type of assistance sought, the pathways used to surrender, and the concerns reported by respondents during surrender. For example, the New Acquisitions class was more likely to relinquish to a shelter, either as the only method or after attempting to self-rehome their animal. The Behavioural Incompatibility class had the highest proportion of participants who attempted to relinquish to a shelter but ended up self-rehoming, and the Owner Hardships class had the highest proportion of participants who self-rehomed as their only method. In addition, the classes varied in the proportion of respondents who reportedly sought assistance to help keep their pet. Qualitative analysis revealed that respondents wanted or sought a variety of different support services, including behavioural support, part-time care, and veterinary care. Future research should consider the heterogeneity in surrender decision-making when addressing issues of intake diversion from animal shelters.

## Introduction

Animal shelters aim to reduce relinquishment to their facilities in order to minimize overall intake, which falls under an umbrella of efforts termed ‘intake diversion’ [1,2]. For owner relinquished animals, one diversion strategy is to suggest other methods of surrender, such as through friends, family, or online [3,4]. Broadly, these methods are termed “self-rehoming” and may include any surrender method outside of shelter relinquishment. Indeed, previous research has demonstrated that some pet owners who relinquish animals to shelters have tried other methods of surrender prior to final relinquishment to a shelter [5,6]. This suggests that pet owners may already choose self-rehoming as their preferred method to find a new home for their pet but may relinquish their animal to the shelter as a secondary option. A previous study analyzed data from a self-rehoming website, where pet owners can post their animals for surrender without use of a shelter or rescue [7]. The study found that animals posted on the website had increased odds of being adopted through the platform (i.e., diverted from shelters) if they had characteristics desired by potential adopters, such as being purebred, smaller, younger, or having positive behavioural characteristics. These findings suggest that, while self-rehoming may successfully divert most animals that are placed online, animals that are not adopted through self-rehoming methods may be relinquished to animal shelters as a last resort.

Although there is evidence that pet owners seek out assistance to help keep their pets, the breadth of pet support services they seek out prior to surrendering a pet remains understudied [5,8]. In previous interviews of relinquishing pet owners, the number of people who pursued assistance to keep their pets was low [6,9]. From animal shelters’ perspectives, another example of a diversion strategy may be to provide pet support services, such as pet food banks, low-cost veterinary care, or behavioural advice, that help the owner keep the animal [8]. Russo and colleagues [8] surveyed animal shelter organizations, and found a number of barriers to providing community support services, including lack of resources and knowledge among pet owners. In addition, organizations believed that offering resources at the time of intake may be too late. Previous surveys that inquired about pre-surrender assistance have focused largely on whether pet owners sought services from animal shelters [5]; however, given that the decision to surrender animals may begin long before the owner connects with the shelter [6], understanding pet owners’ attempts to find support and potential barriers to accessing support may inform more effective intervention strategies.

It remains unclear how pet owners select the method of surrender and what factors may contribute to the decision to surrender an animal through self-rehoming versus a shelter. For example, research has hypothesized various factors that might contribute to the choice of relinquishment over accessing other services, such as mandatory return policies [10], lack of access to support [11], or positive past experiences with animal shelters [5]. On the other hand, other research has suggested that shelter relinquishment may not be the foremost choice for pet owners due to concerns about harm to animals or potential euthanasia [6,12]. Further, previous research has suggested other factors that relate to surrender decisions, such as having other pets in the home, length of ownership, and the source of acquisition [5,13,14]. Typically, when an animal enters the shelter, information about the circumstances leading to surrender (e.g., one or two surrender reasons) are often collected and researched [15–17]; however, they may not reflect the multifactorial decisions that confront an owner [18,19]. While some studies have touched upon aspects of the decision to relinquish animals to shelters [6,11,20,21], there are very few studies dedicated to analyzing surrender from the perspective of pet owners, particularly understanding the relationship between the circumstances that lead to surrender and the choice of surrender method (i.e., relinquishment to a shelter or self-rehoming). In this study, the term surrender refers to any method of transferring pet ownership, while methods of surrender will be specified as either self-rehoming or shelter relinquishment.

Broadly, the goal of this study was to further the literature about relinquishment to shelters and alternative, community-based diversion strategies. However, while previous studies have typically focused on the factors that may contribute to surrender separately [14,22], few studies have attempted to identify groups of pet owners who share similar patterns of characteristics and circumstances leading to surrender. As such, the present study used latent class analysis (LCA) to understand what animal- and owner-related characteristics were likely to co-occur as reported by pet owners. LCA is a modelling technique where the underlying assumption is that membership in unobserved, latent classes can be explained by patterns across indicators (e.g., characteristics, scales; [23]). LCA is used in human health research to understand groups represented by multiple risk factors [24], and interpretation of LCA classes may be beneficial to discourage “one size fits all” approaches to complex issues [25]. In research about companion animals more broadly, LCA has been used to identify typologies of pet owners and non-owners based on demographic and health characteristics [26] and temperament profiles of dogs across breeds [27]; however, this technique has never been applied to animal shelter issues.

Using a sample of U.S. and Canadian public members who rehomed a pet within the past five years, the present study aimed to identify groups of pet owners who share similar patterns of responses to owner-, animal- and decision-related circumstances surrounding their recent surrender. Second, we aimed to compare the identified classes across mixed methods survey questions to reveal differences in method of surrender, assistance sought, and worries during the surrender process.

## Materials and methods

### Data collection

The survey was opened for data collection from January 19 to June 11, 2023. The full survey can be found in S1 File. Public members over 18 years old were recruited to take the survey through convenience sampling. The survey advertisement was shared by the authors through social media across the United States (U.S.) and Canada via rehoming Facebook groups. The survey was also shared through animal shelters and rescues to ask if organizations could advertise via flyers in their lobbies. As well, Facebook advertisements were used to promote the survey, targeting adults who lived in the U.S. or Canada. All procedures were approved by the University of British Columbia’s Behavioural Ethics Review Board (H22-02967).

### Survey

Participants read a brief description of the study and possible risks and provided written consent before proceeding with the survey. The next page showed the screening question (if they rehomed an animal within the past 5 years). The first section asked about the circumstances of their most recently rehomed pet, including how long ago the surrender occurred, the length of ownership prior to surrender, and the type of pet rehomed. For those who rehomed a dog or cat, an additional question appeared asking if the pet was purebred or mixed breed. For individuals who rehomed a dog, an additional question about dog size appeared. All respondents were asked other morphological and behavioural questions about their rehomed animal (e.g., age, sex, needed an experienced adopter, good with children).

The second section asked respondents about the circumstances leading to the surrender, including the surrender reasons, whether they sought and received assistance, whether they felt they had to withhold information about the surrender reason to the person taking their animal, and how long it took for them to come to the decision to surrender. Respondents were asked to select all the surrender methods they had attempted and to select the final successful method of surrender. Categories for surrender methods were divided into those who self-rehomed as their first and only option (SR only), those who attempted to self-rehome then ultimately relinquished to a shelter (SR-RQ), those who relinquished to a shelter as their only option (RQ only) and those who attempted to relinquish to a shelter but then self-rehomed (RQ-SR). Then, respondents were asked, on a 5-point Likert scale (1 = strongly disagree, 5 = strongly agree), to rate their agreement with nine statements that listed various worries that they had when they were surrendering their animal.

Some sections were not included in the present analysis. One section inquired about emotional impact of the surrender process and another asked participants about their general attitudes toward animal shelters. The final section asked participants about their actions following surrender, including whether they acquired another pet and what method they would choose if they surrendered another animal. On the final page, participants were allowed to leave feedback about the survey and describe anything else about their pet surrender that they wanted to share.

## Analysis

### Model indicators

Patterns of responses to questions about the surrender circumstances were identified by conducting LCA [28] through the R package poLCA [29,30]. Twenty-one variables were selected as indicators for the LCA model. The reasons for surrender and animal-related characteristics were included because they were largely aligned with many of the variables collected by animal shelters at the time of intake [19,31]. The indicators included eight categories of surrender reasons (housing issues, animal behaviour issues, cost issues, permanent personal issues, temporary personal issues, having too many animals, owner health issues, and other reasons) which were coded as a binary variable (1 = yes, 0 = no). As well, eight animal characteristics were included in the LCA. Animal-related characteristics included the type of animal (1 = dog, 0 = cat), breed (1 = mixed breed, 0 = purebred), age (1 = adult/senior, 0 = young), sex (1 = male, 0 = female), neutered (1 = not neutered, 0 = neutered), good with children (1 = not good with children, 0 = good with children), good with pets (1 = not good with pets, 0 = good with pets), experienced adopter needed (1 = needed an experienced adopter, 0 = did not need an experienced adopter). Based on previous literature and author experience, the responses to these surrender decision items were coded from the shelters’ perspective, where “more vulnerable” responses (i.e., at a high risk of a poor outcome) coded as 1 and the “less vulnerable” responses were coded as 0 for each indicator [e.g.,^10^,^32^–^34^]. For example, for age, adult/senior animals were coded as 1, while young animals were coded as 0 [35]. Other indicators about the surrender decision were included based on hypothesized relationships to surrender method. This included the animal source (1 = shelter, 0 = non-shelter), length of ownership (1 = less than 1 year, 0 = more than 1 year), length of the surrender decision (1 = less than 1 month, 0 = greater than 1 month), and whether there were other pet(s) in the home at the time of surrender (1 = yes, 0 = no). Finally, as pet owners in this survey may have rehomed an animal up to five years ago, the timing of the surrender was included (1 = more than 1 year ago, 0 = less than 1 year ago).

### Model estimation and selection

The number of classes was increased, beginning with the 1-class solution, until the best fitting model was identified [23,29]. Best fit was determined by assessing Pearson’s χ2 goodness of fit and likelihood ratio chi-square (G2) statistics, Akaike information criteria (AIC), Bayesian information criteria (BIC), adjusted BIC and corrected AIC, where smaller values indicate better model fit [36,37]. In addition to fit statistics, we also considered the proportion of respondents that were represented in each class and the theoretical meaningfulness of the classes [23,38]. Once the best fitting model was chosen, class membership was assigned based on the highest probability for each respondent [29].

### Class membership and surrender process

Relationships between class and surrender process items (i.e., whether the pet owner sought assistance, the surrender methods used) were assessed through the Fisher’s Exact Test with the Cramer’s V reported for effect size. Then, the Kruskal-Wallis test was used to assess the relationship between class membership and participants’ responses to the questions about the worries when they were surrendering. Due to multiple comparisons, the *p*-values were adjusted with the Bonferroni correction [39]. Values of *p* < 0.05 were considered statistically significant.

For one open-ended question “Why did you/did you not seek assistance to help you keep your pet? If you did seek assistance, what type of assistance was it?”, responses were qualitatively coded using inductive thematic analysis [40], then compared across assigned classes. For the purposes of this study, thematic analysis focused on the type of assistance that was wanted or sought by respondents. First, the first author (LL) read through the responses and created a preliminary codebook. Then, two trained research assistants individually coded the same 30 responses using the codebook. These researchers then discussed the results and edited the codebook based on discrepancies. This process was repeated for six sets of 30 responses until a final codebook was agreed upon. Then, researchers independently coded approximately 20% of the responses at a time with the final codebook, each time discussing and resolving any remaining discrepancies. Responses could contain multiple qualitative themes and are summarized descriptively by class.

For questions where respondents were able to select “Other” and write their own response, the written responses were also recoded into existing categories or into created categories through the same process as described, although with pre-existing code themes from the multiple-choice answers.

## Results

### Survey participants

The total number of participants who began the survey was 1292. Participants were removed for incomplete surveys (n = 454), not meeting the survey criteria (n = 200), and failing the attention checks (n = 92). Some participants (n = 29) provided non-intelligible, one-word qualitative responses or had duplicated qualitative responses that suggested bot activity and were also removed. The final sample consisted of 517 participants. The sample was approximately evenly split between Canada (47.0%, n = 243) and the U.S. Canadian respondents mostly lived in British Columbia (24.4%, n = 126), Ontario (9.7%, n = 50) and Alberta (4.1%, n = 21). From the U.S., 105 respondents were from the West census region (20.3%), 84 from the South region (16.2%), 48 from the Midwest region (9.3%), and 37 from the Northeast region (7.2%). The mean (± SD) age of respondents was 38.9 (± 14.6) years. Most respondents identified as women (76.0%, n = 393), while the rest identified as men (17.6%, n = 91), non-binary (4.4%, n = 23) or preferred not to say (1.9%, n = 10). Most of the sample was also employed in some manner (67.3%, n = 350), while the rest of the respondents were homemakers, students, retired, and otherwise receiving assistance (28.2%, n = 143), or unemployed (4.1%, n = 21).

Among all survey participants, the most surrendered animals were dogs (54.2%, n = 28), followed by cats (33.3%, n = 172). Only 12.6% (n = 65) had surrendered a different type of pet (“Other”), including small animals (e.g., hamster, rabbit; 6.6%, n = 34), large animals (e.g., horse; 1.4%, n = 7), and exotic animals (e.g., bird, lizard; 4.6%, n = 24). All “Other” species were removed for subsequent analysis, leaving 280 dogs and 172 cats.

### Classes of surrender characteristics

**Fig 1:**
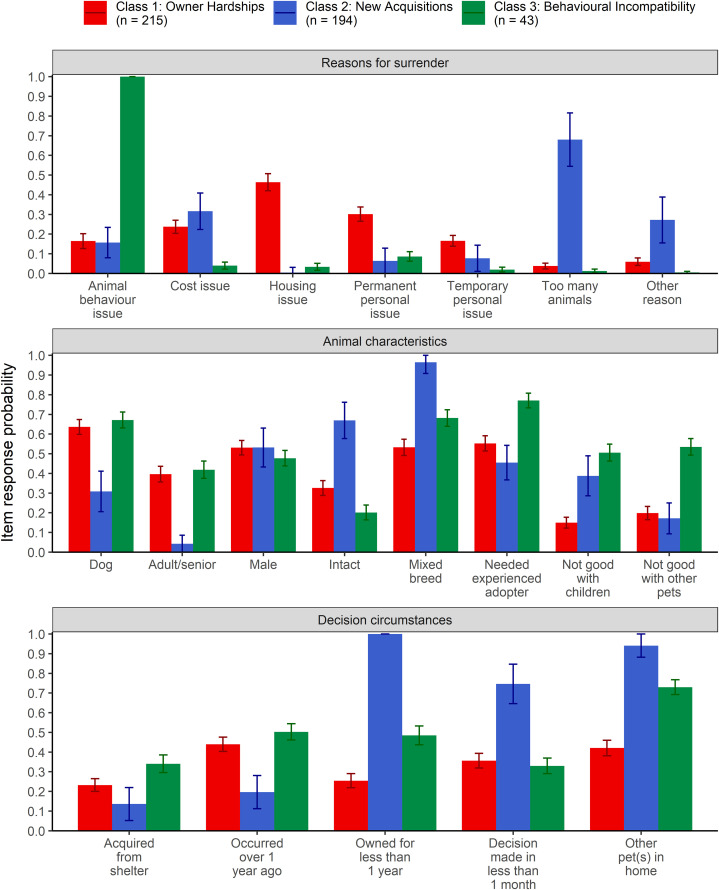
Item-response probabilities with standard error for reasons for surrender, animal characteristics, and decision circumstances separated by the three-class LCA model. Participants (n = 452) were recruited from the U.S. and Canada and reported based on the most recent dog or cat that they surrendered (through self-rehoming or shelter relinquishment) within the past five years.

The LCA identified a three-class model of surrender characteristics as the best fit (Table 1). The best-fitting model showed high entropy (i.e., high separation of classes); as such, class membership with the highest probability was assigned to each respondent and used to explore differences across classes [29]. Fig 1 shows the three-class model and item-response probabilities. The three classes showed a high degree of separation based on reasons for surrender and length of ownership. As such, the three classes were named *Owner Hardships* (Class 1; n = 215, 47.6%), *New Acquisitions* (Class 2; n = 194, 42.9%), and *Behavioural Incompatibility* (Class 3; n = 43, 9.5%).

**Table 1:** Fit statistics for unconditional latent class analysis for models with 1 to 6 classes (AIC = Akaike information criterion; BIC = Bayesian information criterion). The final solution is bolded. Participants (n = 452) were recruited from the U.S. and Canada and reported based on the most recent dog or cat that they surrendered (through self-rehoming or shelter relinquishment) within the past five years.

Number of classes	Max log-likelihood	AIC	BIC	Adjusted BIC	Corrected AIC	Entropy
1	−5278.08	10598.16	10684.55	10617.9	10705.55	–
2	−5108.14	10302.29	10479.17	10342.71	10522.17	0.818
**3**	**−4989.07**	**10108.14**	**10375.53**	**10169.25**	**10440.53**	**0.844**
4	−4926.04	10026.08	10383.97	10107.87	10470.97	0.807
5	−4869.25	9956.497	10404.89	10058.96	10513.89	0.802
6	−4826.32	9914.629	10453.52	10037.78	10584.52	0.804

### Class 1: Owner Hardships

Participants in the Owner Hardships class had a higher probability of indicating that housing-related issues contributed to surrender (probability ± SE = 0.46 ± 0.04), and a slightly higher probability of indicating that permanent personal issues (0.30 ± 0.04) and temporary personal issues (0.17 ± 0.03) contributed to surrender compared to the other two classes. As well, the Owner Hardships class had a higher probability of surrendering a dog (0.64 ± 0.04) and surrendering an adult/senior animal (0.40 ± 0.04) compared to the New Acquisitions class but had similar probability for these variables compared to the Behavioural Incompatibility class.

### Class 2: New Acquisitions

Participants in the New Acquisitions class had a higher probability of indicating that having too many animals contributed to surrender (0.68 ± 0.14) and a slightly higher probability of indicating that cost-related issues contributed to surrender (0.32 ± 0.09) compared to the other two classes. Additionally, participants in this class had a higher probability of owning the animal for less than a year (1.00 ± 0.01) and making the decision to surrender their animal in less than 1 month (0.75 ± 0.10). This class also had a higher probability of having other pets in the home at the time of surrendering (0.94 ± 0.06). The probability of surrendering a male pet was higher (0.53 ± 0.10) than the Owner Hardships class, but not the Behavioural Incompatibility class. The probability of surrendering an intact animal was higher in the New Acquisitions class (0.67 ± 0.09) compared to the Behavioural Incompatibility class.

### Class 3: Behavioural Incompatibility

Participants in the Behavioural Incompatibility class had a higher probability of indicating that issues with their pet’s behaviour contributed to surrender (1.0 ± 0.00) compared to the other two classes. In comparison to the Owner Hardships class, participants in the Behavioural Incompatibility class had a higher probability of surrendering pets that were not good with other animals (0.54 ± 0.04) and not good with children (0.51 ± 0.04). In comparison to the New Acquisitions class, the Behavioural Incompatibility class had a higher probability of surrendering a dog (0.67 ± 0.04) and an adult/senior animal (0.42 ± 0.04).

### Class membership and surrender process

#### Pet retention assistance.

Respondents were asked whether they sought any assistance that would help them keep their pet. Table 2 shows the number and proportion of respondents that reported seeking assistance to help keep their pet, as well as the surrender methods used, separated by class. Fisher’s Exact Test revealed a difference between classes and seeking assistance (*p* < 0.001, Cramer’s V = 0.23). The New Acquisitions class had the highest proportion of participants report that they sought assistance to help keep their pets (n = 106, 54.6%), followed by the Owner Hardships class (n = 98, 45.6%). On the other hand, very few in the Behavioural Incompatibility class sought assistance to help keep their pets (n = 6, 14.0%).

**Table 2:** Number and proportion of participants who reported seeking assistance and using each surrender pathway for an animal that was surrendered within the past five years (n = 452), categorized as those who relinquished their animal to a shelter as their only option (RQ only), those who tried to relinquish to a shelter, but ended up self-rehoming (RQ-SR), those who self-rehomed as their only option (SR only), and those who tried to self-rehome their animal, but then ended up relinquishing to a shelter (SR-RQ). The data are separated based on the class assignment of a three-class LCA model.

Variable	Level	Class 1: Owner Hardships (n = 215)		Class 2: New Acquisitions (n = 194)		Class 3: Behavioural Incompatibility (n = 43)		Overall	
		n	%	n	%	n	%	n	%
**Sought assistance**									
	Yes	98	45.6%	106	54.6%	6	14.0%	210	46.5%
	No	117	54.4%	88	45.4%	37	86.0%	242	53.5%
**Surrender method**									
	RQ only	17	7.9%	36	18.6%	4	9.3%	57	12.6%
	RQ-SR	42	19.5%	32	16.5%	17	39.5%	91	20.1%
	SR only	140	65.1%	107	55.2%	20	46.5%	267	59.1%
	SR-RQ	16	7.4%	19	9.8%	2	4.7%	37	8.2%

Qualitative analysis revealed six types of assistance that were wanted or sought by pet owners during the surrender process, which are shown separated by class in Table 3. The themes are described briefly with example quotes (shown with the participant number and assigned class).

**Table 3:** The frequency of each qualitative theme discussed in response to the open-ended question “If you did seek assistance, what type of assistance was it?” The data are separated based on the class assignment of a three-class LCA model. Participants (n = 452) reported on the most recent dog or cat that they rehomed within the past five years. Responses may have been coded under multiple themes, so the percentages may add up to more than 100.

Theme	Class 1: Owner Hardships (n = 215)		Class 2: New Acquisitions (n = 194)			Class 3: Behavioural Incompatibility (n = 43)			Overall			
	n	%	n	%		n	%		n		%	
**Type of assistance wanted/sought**	**55**	**25.6%**	**94**	**48.5%**		**7**	**16.3%**		**156**		**34.5%**	
Behavioural support	7	12.7%	75	79.8%		2	28.6%		84		53.8%	
Part-time care	27	49.1%	11	11.7%		1	14.3%		39		25.0%	
Veterinary care	5	9.1%	25	26.6%		2	28.6%		32		20.5%	
Human support	11	20.0%	3	3.2%		2	28.6%		16		10.3%	
Animal shelter support	4	7.3%	4	4.3%		1	14.3%		9		5.8%	
General pet supplies	4	7.3%	2	2.1%		0	0.0%		6		3.8%	

*Behavioural support*: Assistance sought to improve behavioural issues in pets ranged from at-home methods (e.g., “changes in environment, additional litter boxes, new food” (P606; New Acquisitions)) to professional help “We worked with a dog trainer for 6+ months to help with behavioural issues” (P143; New Acquisitions). In some cases, the behavioural assistance sought was extensive and to no avail, “I worked with several trainers and a behaviorist for years. [My pet] and I were like oil and water trying to mix” (P15; New Acquisitions).

*Part-time care:* Part-time care came in the form of both daily care and longer-term care for pets. The majority of the respondents in this theme sought part-time care from family or friends (e.g., “I had friends stay with him but then I ran out of friends to stay with him” (P416; Owner Hardships), “My parents were able to keep him until I could find an appropriate home for him” (P1094; Owner Hardships). In some cases, respondents used professional services, for example, “I used dog walkers and other services that would provide him with entertainment, but eventually couldn’t afford them on the regular basis that was needed” (P244; Owner Hardships). In two cases, respondents used temporary boarding services offered by animal shelter organizations.

*Veterinary care:* Most often participants sought recommendations or medications from veterinarians to help with behavioural issues, for example, “[I] discussed with [a] vet and tried medication, but [the behaviour] was escalating and was unsafe for our kids.” (P250; New Acquisitions). Less commonly, respondents stated that the veterinarian was sought for medical treatments. For example, one participant said, “her medical treatment would have cost clos[e] [*sic*] to $1,000 and could have possibly been on going” (P571; Behavioural Incompatibility).

*Human support:* The owner-related circumstances with which respondents needed assistance included financial support, housing, human health, and lifestyle changes. Some respondents described cost or time issues with accessing the assistance they would need (e.g., “I tried every option I could search for to help find housing that would allow me to keep my cats and simply ran out of time” (P153; Owner Hardships), “We can’t afford to move to a place with a bigger yard” (P323; New Acquisitions)). A few respondents described health or lifestyle changes they made to accommodate their pet. One individual said they “worked with [their] employer to adjust [their] hours” (P704; New Acquisitions) in an effort to keep their pet. In dealing with allergies to pets, one respondent said, “I sought out medical treatment to try and treat the allergies” (P609; New Acquisitions).

*Animal shelter:* Some individuals in this theme discussed contacting the shelter but did not elaborate on the type of assistance sought. One individual visited a pet food bank. The other type of assistance discussed was advice or resources, “[the shelter] gave advice, but I was already doing all they suggested” (P263; New Acquisitions). One individual connected with the behavioural coordinator from a shelter for assistance, “I was not made aware of her behavioral or medical issues before adopting and the behavioral coordinator from the shelter came out to help and divulged all the issues the pet actually had” (P248; New Acquisitions).

### Surrender methods

Fisher’s Exact Test showed a difference between class membership and surrender methods (*p* = 0.002, Cramer’s V = 0.16; Table 3). The New Acquisitions class had the highest proportion of participants who relinquished their animal to a shelter as their only method (RQ only; 18.8%) and attempted to self-rehome their animal but ended up relinquishing their animal to a shelter (SR-RQ; 10.2%). The Behavioural Incompatibility class had the highest proportion of participants who attempted to relinquish to a shelter but ended up self-rehoming (RQ-SR; 33.3%). The Owner Hardships class had the highest proportion of participants who self-rehomed as their only method (SR only; 64.1%).

### Worries during surrender

**Fig 2:**
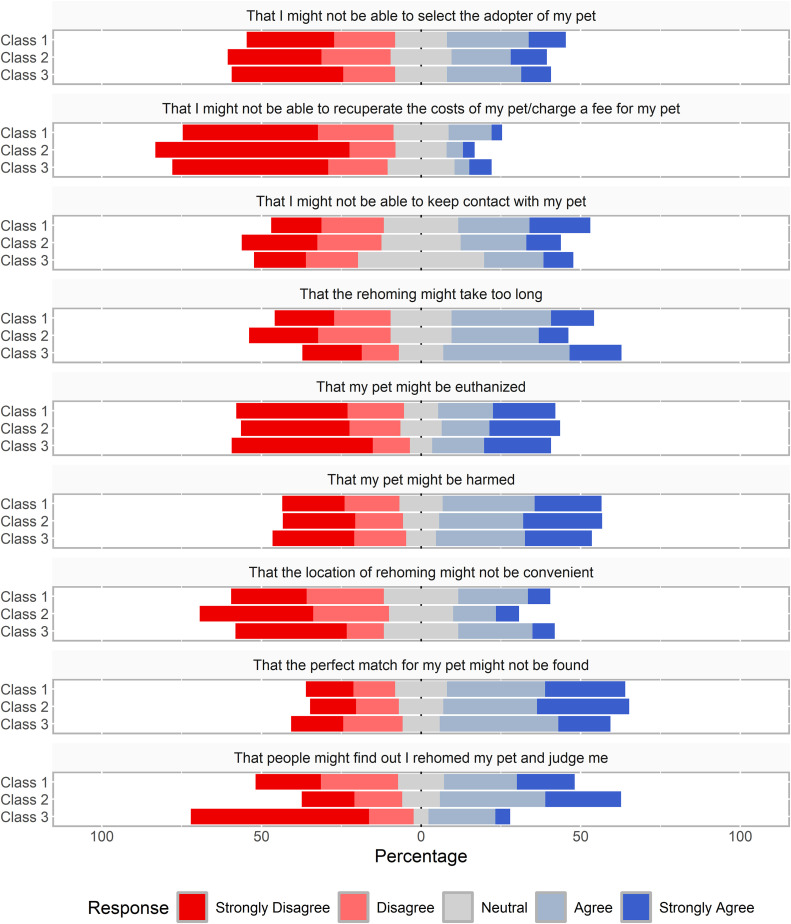
Participant agreement on a five-point Likert scale (Strongly Disagree to Strongly Agree) in response to multiple statements about worries during the surrender (i.e., self-rehoming or shelter relinquishment) of a pet within the last five years (n = 452). The data are separated based on the class assignment of a three-class LCA model (Class 1: Owner Hardships = 215, Class 2: New Acquisitions = 194, Class 3: Behavioural Incompatibility = 43).

Fig 2 shows the Likert responses to questions about worries that participants faced when surrendering their animal, separated by classes. Kruskal-Wallis tests revealed a difference in Likert responses to the item that described worry about being able to recuperate costs of their pet/charge a fee for their pet (*p* = 0.02). Participants in the New Acquisitions class reported lower mean (±SD) agreement (1.8 ± 1.1) compared to the other classes (Owner Hardships = 2.1 ± 1.2, Behavioural Incompatibility = 2.1 ± 1.2). In addition, there was a difference in Likert responses to the item that described the worry about judgement regarding their decision to surrender. Participants in the New Acquisitions class reported higher mean agreement (3.3 ± 1.4) compared to the other classes (Owner Hardships = 3.0 ± 1.4, Behavioural Incompatibility = 2.3 ± 1.4).

## Discussion

This study explored segmentation of pet owners who rehomed an animal up to five years ago based on their reasons for surrender, animal characteristics, and decision-making circumstances. LCA indicated what animal- and owner-related characteristics were likely to co-occur, as reported by pet owners, and allowed for exploration of differences in surrender pathways and worries between subpopulations of pet owners. The results revealed three classes of participants that were most distinctly distinguished by the reasons that contributed to surrender and the length of ownership. Comparison of outcomes across classes suggested that subgroups may seek different assistance, attempt and succeed at different surrender methods, and report different worries about the surrender process. Overall, this is the first study that has applied the LCA method to data about companion animal surrender circumstances.

## Classes of surrender characteristics

The reasons for surrender explored in the current study were based on categories that are similar to that of previous research [e.g.,^17^,^41^], as well as recommended by national organizations [42]. Analysis of data collected at intake typically separates reasons into general categories (e.g., owner-related and animal-related reasons) [41] or by analyzing outcomes by individual reason [e.g.,^14^]. However, the results of the LCA suggest that decisions and circumstances leading to surrender may be more nuanced than typically collected and reported in prior studies. The present study suggested that indicators beyond surrender reasons (e.g., length of ownership, household pets) may also be important to define types of surrendering pet owners that may benefit from different support. For example, pet owners reflected in the New Acquisitions class were defined owning the animal for less than a year, but also other circumstances (e.g., Too Many Animals, other pets in home) and animal-related characteristics (e.g., intact animals, mixed breed animals, young/juvenile animals) that may indicate incompatibilities with other household pets or unwanted litters. For this dataset, LCA provided a useful tool to identify underlying subgroups based on multiple observed characteristics, which may suggest different avenues for support. Given that the present study used a convenience sample, future studies should aim to test whether these discrete classes of people who endorse similar patterns of surrender characteristics emerge in representative samples.

### Class membership and surrender process

Comparisons across the three classes suggested that surrender circumstances were related to assistance-seeking behaviours and the pathways participants took to surrender. However, findings related to the Behavioural Incompatibility class should be interpreted with caution due to its relatively small size (n = 43), which may reduce the statistical power of estimates for this group. Given the clear separation between classes, satisfactory model fit, and meaningful interpretability, comparisons across classes were retained.

Comparing the number of participants who sought assistance across classes revealed that the Behavioural Incompatibility class had the smallest proportion of pet owners who sought assistance. This may indicate particular barriers with seeking assistance for animal behaviour issues, such as cost, accessibility, or commitment. In interviews with owners who relinquished to a shelter, Irvine [9] found that few people who surrendered dogs for aggression had attempted to seek help from behaviourists or veterinarians. In the United Kingdom, Diesel and colleagues [14] similarly reported that within relinquishing dog owners who reported problematic behaviours, 69.2% did not seek any behavioural advice to help keep their dog. In our study, behavioural support was sought more often from trainers or personal networks rather than animal shelters. Russo and colleagues [8] found that around one third of surveyed U.S. organizations provided behaviour consultations or training classes, while others were often unable to provide these services due to resource constraints. The authors stated that it is unclear whether community members would view shelters as a resource for behavioural support. In addition, one study in the U.K. found that around one-quarter of relinquishing pet owners accepted free behavioural advice offered by the animal shelter [43]. Our study supports the notion that, while affordable behavioural services are important to reduce the need for pet surrender, owners do not necessarily view sheltering organizations as resources for pet behaviour support. Organizations may consider partnering with community services (e.g., veterinary clinics, animal behaviour professionals) to identify families at risk of surrender and provide proactive support.

Qualitative responses also suggested that the support that was sought did not necessarily reflect the rehome reason with the highest probability within each class, suggesting multifaceted circumstances. For example, the New Acquisitions class had the highest proportion of responses coded under *behavioural support*. This may suggest that the reason for surrender that is reported may not always be fully reflective of the circumstances. In addition, the New Acquisitions group had a higher proportion of relinquishments to the shelter, either as the only option or after attempting to self-rehome the animal (RQ only, SR-RQ). Interestingly, while this class was likely to make the decision to rehome their animal within a short period of time (less than one month), they also had the highest proportion of respondents report that they sought assistance to help keep their animal. It is possible that the New Acquisitions class sought support to transition a new pet into their home. On the other hand, this may suggest that owners who exhaust other means of assistance may then bring their animal to a shelter, which was reported in previous research [6]. This has implications for the success of support services offered at the time of relinquishment to a shelter, as previous accounts from animal shelter staff suggested that one barrier to implementing intake diversion services was that pet owners did not want or need the services at the time of relinquishment [8,44].

Notably, the Owner Hardships class had the highest proportion of responses coded under *part-time care,* mostly in the form of temporary care during a crisis or help with day-to-day care from professionals or personal networks. Some shelters provide temporary boarding for owners who are undergoing a crisis, such as fleeing domestic violence or hospitalization [e.g., 45]; however, shelters typically do not help with day-to-day care of animals. Most often, part-time care was sought from social support networks, like friends or family. This finding may suggest that future research is needed to understand the role of social support networks to support pet ownership. As pet care more becomes closer to care of human children among families [46], it may be important to consider how lack of access to pet care may mirror the childcare crisis [47,48]. Investigating stressors during the COVID-19 pandemic, Applebaum and colleagues [49] found that 9% of survey respondents cited concerns about finding friend, family, or professional care for pets in the event of falling ill. Indeed, they concluded that sole responsibility for pets could increase stress during times of crises. Given that many individuals in the present study would have required temporary or day-to-day care to retain their animal, more research could be dedicated to services such as temporary fostering or cooperative (e.g., multi-family) pet ownership.

The present analysis revealed differences in proportions of respondents who tried surrender pathways across classes. Overall, most respondents in our survey had surrendered an animal through self-rehoming methods, which potentially suggests that the practice of self-rehoming is common. Indeed, previous studies in shelters found between 45–85% of owners, who relinquished an animal to a shelter, had previously attempted to self-rehome their animal [5,6,50]. Our study also found that approximately 40% of the RQ only group tried at least one self-rehoming method before relinquishing their pet. Future research may consider investigating the overall prevalence of self-rehoming and relinquishment in the pet-owning population through more representative sampling.

Our results are somewhat concurrent with previous literature that suggests that surrender reasons may be associated with the method of surrender used by pet owners. One previous study by Weiss and colleagues [11] conducted a random digit dial survey about surrender in the U.S., and found that those who were rehomed for a behavioural issues were more likely to be relinquished to a shelter, while those who rehomed for family and housing issues were more likely to be rehomed to family or friends. Similarly, our study found that the Owner Hardships class had the highest proportion of individuals who self-rehomed as their first and only option. In contrast, the present study found that individuals in the Behavioural Incompatibility class reported the highest proportion of individuals who attempted to relinquish to a shelter but were ultimately self-rehomed (RQ-SR). It is possible that this group of surrendering pet owners represents those who were diverted from animal shelters. This result may not align with previous findings that suggested that owners surrendering their dogs for behavioural reasons were less likely to successfully find a new home through a self-rehoming platform (and therefore were more likely to relinquish their animal to a shelter instead [7]. However, it remains unclear if the unsuccessful relinquishment to the shelter was due to provision of support services that enabled diversion, or other hypothesized situations that might result from deferring intake (e.g., could not wait for shelter appointment, did not want to follow through with relinquishment) [1]. Future research should consider investigating the process of diversion at the time of intake by following up with pet owners who connect with animal shelters during the surrender process.

Comparison of class membership and responses to questions about worries during the surrender process revealed that the New Acquisitions class were the least worried about recuperating costs for their rehomed pet. The New Acquisitions class also had the highest proportion of respondents who relinquished to a shelter, where pet owners are unable to charge a fee to rehome (and in some cases, may be asked to pay a fee to relinquish). Therefore, these responses may reflect the final surrender method that participants used, rather than the characteristics of the classes themselves. Further, the New Acquisitions class agreed more strongly with the worry about judgement regarding the decision to rehome their animal. Broadly, research has suggested that relinquishment to shelters is stigmatized, largely due to the institutional and societal belief that pets (particularly dogs and cats) are a lifetime commitment [9]. One study of U.S. post-secondary students found that participants generally found relinquishment to be unacceptable; however, attitudes toward scenarios that described different reasons for relinquishment differed [51]. Participants reported more sympathy for those relinquishing for cost-related issues or serious dog behavioural problems, while scenarios that outlined housing-related issues or nuisance behavioural issues were considered less acceptable. Relinquishing for having an excess of animals was not tested in this study, and all the scenarios ended in shelter relinquishment. Future studies may consider understanding stigma faced by pet owners, comparing both surrender reasons and methods, to understand potential impacts on pet owners, as well as identify how animal shelters and community members can improve the process of relinquishment and diversion.

### Limitations and future directions

There are several limitations to the present study. Foremost, data were collected through an online convenience sample, which may have led to selection bias toward positive surrender experience, those who have access to technology, and those who are more willing to share their experiences. Previous studies that purposefully sampled in shelters also reported some degree of sampling bias. For example, Digiacomo and colleagues [6] found that 11 out of 49 relinquishing pet owners declined to be interviewed, some stating that they were too upset to participate, which may have produced a similar bias toward more positive experiences.

The present findings are exploratory, and thus, the class structure and relative proportions may not be representative of the entire population of surrendering pet owners. The LCA model is bounded by the set of indicators specified a priori based on previous literature about owner surrender; therefore, the absence of certain constructs in the classes may reflect limitations of the questions regarding the circumstances that lead to surrender. A future study should validate the class structure in a representative sample. This study inquired about surrender events that may have occurred up to five years ago, which may impact how individuals retrospectively viewed the circumstances leading to surrender, particularly due to the sensitive nature of pet rehoming [6]. Rehoming occurrence was included in the present LCA model. The Behavioural Incompatibility class was slightly more likely to have surrendered their animal over one year ago compared to the New Acquistions class. There were no other differences in the item response probabilities for rehoming occurrence between the three classes. With the current data, it is unclear whether this is due to recall bias or due to environmental changes that may impact reasons for surrender over time. For example, one study in British Columbia found that between 2008 and 2019, the proportion of dogs relinquished to animal shelters due to the reason of having too many animals and behavioural concerns increased, while the proportion of dogs relinquished due to financial concerns decreased [17]. Future research may consider methods to reduce recall bias, such as sampling pet owners who surrendered an animal within a more recent time frame or utilizing case-control design.

Future research should consider parsing the chronology of the surrender decision. In the present study, all surrender methods were grouped into self-rehoming or relinquishing, so the order of the individual surrender methods and multiple attempts at these methods are not considered in our analysis. Finally, the qualitative question did not specify the criteria for what was considered “seeking”; consequently, responses to this question may reflect various degrees of assistance (e.g., considering, searching, receiving assistance). As such, responses were coded to capture broadly the types of assistance that were wanted or sought. Future research may consider understanding pet surrender using methodologies that capture the timeline leading to surrender. For example, a faux longitudinal research design could gather data related to an extended period of time retrospectively through reconstruction of events [52].

## Conclusion

The present study found that different patterns of reasons for surrender, decision circumstances, and animal characteristics may be associated with differences in seeking pet support services and pathways of surrender. Pet owners who were experiencing owner hardships were more likely to need assistance with part-time care and engaged in self-rehoming, while those who surrendered due to behavioural incompatibilities with their pets did not seem to seek assistance and ended up self-rehoming even if trying to surrender to an animal shelter first. Finally, pet owners who surrendered shortly after acquisition were more likely to seek out behavioural support and surrendered directly to an animal shelter. Future research about intake diversion should consider the variation in circumstances and pathways that lead to animal shelter relinquishment in order to create more effective interventions to encourage pet retention.

## Supporting information

S1 File Survey questions for Ly et al., 2024. Online survey that was completed by pet owners in the U.S. and Canada who had surrendered an animal within the past five years. The survey began after the participants read and agreed to the consent form.
(DOCX)
