# Supplementary material for: Support-seeking and rehoming pathways differ by surrender circumstances among pet owners
Source: PLoS One. 2026 Mar 30;21(3):e0345326. doi: 10.1371/journal.pone.0345326 (PMC13035137; doi:10.1371/journal.pone.0345326)
Supplement: S1 File — Online survey that was completed by pet owners in the U.S. and Canada who had surrendered an animal within the past five years. The survey began after the participants read and agreed to the consent form. (DOCX) [file pone.0345326.s001.docx]

**Introduction**

The purpose of this survey is to learn more about the emotional impacts of pet rehoming. You will be asked to recall a time when you rehomed an animal (i.e., gave a pet away to another family or surrendered a pet to an animal shelter or rescue). We understand that the circumstances that lead to rehoming and experiences of rehoming greatly differ across pet owners and variation in emotion is expected. Some people might find rehoming a very difficult experience, while others might not. There are no right or wrong answers as emotions are individual and are never wrong.

We very much value your honesty while you answer these questions. We also want to remind you that your responses are anonymous.

Thank you!

Q1 Have you rehomed a pet in the past 5 years?

- Yes (1)
- No (2)

End of Block: Introduction

Start of Block: Rehomed pet demographics

Q2 For the following questions, **think about the pet that you most recently rehomed.**

Q3 How long ago did you rehome your pet?

- Less than 1 month ago (1)
- 1 - 6 months ago (2)
- 6 months to a year ago (3)
- 1 - 2 years ago (4)
- 2 - 5 years ago (5)

Q4 How long did you have your pet prior to rehoming?

- Less than 2 weeks (1)
- 2 weeks - 1 month (2)
- Around 1 - 6 months (3)
- Around 6 months - 1 year (4)
- 1 - 2 years (5)
- 2 - 4 years (6)
- 4 - 6 years (7)
- More than 6 years (8)

Q5 What type of pet did you rehome?

- Dog (domestic) (1)
- Cat (domestic) (2)
- Small animal (e.g., rat, guinea pig, rabbit, hamster, gerbil, mouse) (3)
- Exotic animal (e.g., bird, reptile, fish, amphibians, hybrids, arachnids, other wild species) (4)
- Large animal (e.g., horse, miniature pig, donkey, alpaca, llama, cow) (5)
- Other (6) __________________________________________________

Display This Question:

If What type of pet did you rehome? = Dog (domestic)

Or What type of pet did you rehome? = Cat (domestic)

Q6 Was your pet purebred or mixed breed?

- Purebred (1)
- Mixed breed (2)

Display This Question:

If What type of pet did you rehome? = Dog (domestic)

Q7 How large was your dog?

- Small (less than 29lbs/13kg. Examples: Miniature Poodle, Pug, Chihuahua) (1)
- Medium (30lbs/13.5kg to 54lb/24.5kg. Examples: Corgi, Australian Cattle Dog, English Bulldog) (2)
- Large (more than 55lbs/25kg; Examples: Labrador Retriever, Golden Retriever, German Shepherd Dog) (3)

Q29 What was the age of your pet at the time of rehoming? Write below in years.

Example: 
Three months old = 0.25
One and a half years old = 1.5
Four years old = 4

________________________________________________________________

Q9 What sex was your pet?

- Female (1)
- Male (2)

Q10 Was your pet spayed/neutered at the time of rehoming?

- Yes (1)
- No (2)

Q11 Was your pet good with kids?

- Yes (1)
- No (2)
- I don't know (3)

Q12 Was your pet good with other pets?

- Yes (1)
- No (2)
- I don't know (3)

Q13 Try to remember the time when you were deciding to rehome your pet. Did your pet have special behavioural or medical needs?

- Yes, my pet had special behavioural needs (e.g., aggression) (1)
- Yes, my pet had special medical needs (2)
- No, my pet did not have special needs (3)

Q14 Try to remember the time when you were deciding to rehome your pet. Did you think your pet required an experienced adopter?

- Yes (1)
- No (2)
- Maybe (Explain) (3) __________________________________________________

Q15 Where did you obtain this pet?

- Pet store (1)
- Breeder (2)
- Shelter/rescue (3)
- Friend/family/personal networks (e.g., acquaintances, co-workers) (4)
- Online advertisement (e.g., social media, Craigslist) (5)
- Pet-specific website (e.g., Adopt a pet, Petfinder) (6)
- Other (7) __________________________________________________

End of Block: Rehomed pet demographics

Start of Block: Rehomed pet - Process

Q15 For the following questions, **think about the pet that you most recently rehomed.**

Q21 **Try to remember the time when you were deciding to rehome your pet.**

What circumstances led to rehoming of your pet? Select all that apply.

- Pet showed aggression (toward human or animal) (1)
- Biting incident (toward human or animal) (2)
- Pet had undesired behaviours (e.g., escaping, destructive, barking, anxiety, not housetrained) (3)
- Couldn’t afford general care (e.g., food, grooming) (4)
- Couldn’t afford pet’s medical expenses (e.g., veterinary visit, spay/neuter) (5)
- Moving and couldn’t take pet (6)
- Couldn't find pet-friendly housing (7)
- Landlord did not allow pets (8)
- Temporary personal issue (e.g., hospital stay, treatment center) (9)
- Permanent life change (e.g., divorce, new baby, relation split) (10)
- Had too many animals (11)
- Other (12) __________________________________________________

Q19 Which methods of rehoming did you try? Select all that apply.

- Tried to return the pet to the breeder (1)
- Tried to rehome through personal networks (e.g., family, friends, acquantainances, co-workers) (3)
- Tried to rehome through online advertisement (e.g., social media, Craigslist) (4)
- Tried to rehome through a pet-specific rehoming website (e.g., Adopt a pet, Petfinder) (5)
- Contacted a shelter or rescue through phone or email for help (6)
- Tried to bring the pet to a shelter or rescue (e.g., a physical facility) (7)
- Other (8) __________________________________________________

Q16 Did you try to seek assistance from others so that you could keep your pet? (For example, finding temporary boarding for your pet, behavioural support, visiting a pet food bank, etc.)

- Yes (1)
- No (2)

Display This Question:

If Did you try to seek assistance from others so that you could keep your pet? (For example, finding... = Yes

Q17 Did you receive the assistance that you were looking for?

- Yes (1)
- No (2)

Q18 Explain your response above. Why did you/did you not seek assistance to help you keep your pet? If you did seek assistance, what type of assistance was it?

________________________________________________________________

Q20 What was the final method that you used to rehome your pet?

- Returned the pet to the breeder (1)
- Rehomed through personal networks (e.g., family, friends, acquaintances, co-workers) (3)
- Rehomed through online advertisement (e.g., social media, Craigslist) (4)
- Rehomed through a pet-specific rehoming website (e.g., Adopt a pet, Petfinder) (5)
- Brought the pet to a shelter or rescue (e.g., a physical facility) (7)
- Other (8) __________________________________________________

Q48 **Try to remember the time when you were deciding to rehome your pet.**

How strongly do you agree with the following statement:

I withheld information about the circumstances that led to the rehoming of my pet (e.g., when writing the online profile, when discussing with friends/family, when talking to shelter/rescue staff)

- Strongly disagree (1)
- Disagree (2)
- Neutral (3)
- Agree (4)
- Strongly agree (5)

Q22 Approximately how long did it take for you to come to the decision to rehome?

- A few days (1)
- 1 - 2 weeks (2)
- 2 - 4 weeks (3)
- 1 - 2 months (4)
- Less than half a year (5)
- More than half a year (6)
- More than a year (7)

Q23 Did you keep contact with the new owners of your pet?

- Yes, and I wanted to (1)
- Yes, but I did not want to (2)
- No, but I wanted to (3)
- No, and I did not want to (4)

Display This Question:

If Did you keep contact with the new owners of your pet? = Yes, and I wanted to

Or Did you keep contact with the new owners of your pet? = Yes, but I did not want to

Q24 How often do you connect with the new owners of your pet?

- About once a year or less (1)
- A few times a year (2)
- About once a month (3)
- A few times a month (4)
- Once a week or more (5)

Q25 Out of all of the things we may be worried about when rehoming our pet, how worried were you about each of these factors? "I was worried …" (1 = Strongly Disagree, 5 = Strongly Agree)

That I might not be able to select the adopter of my pet (1)

That I might not be able to recuperate the costs of my pet/charge a fee for my pet (2)

That I might not be able to keep contact with my pet (3)

That the rehoming might take too long (4)

That my pet might be euthanized (5)

That my pet might be harmed (6)

That the location of rehoming might not be convenient (7)

That the perfect match for my pet might not be found (8)

That people might find out I rehomed my pet and judge me (9)

Q26 Explain your responses above. What were your greatest worries when rehoming your pet? Did you have other worries that were not captured by the options above?

________________________________________________________________

________________________________________________________________

________________________________________________________________

________________________________________________________________

________________________________________________________________

End of Block: Rehomed pet - Process

Start of Block: Pet Bereavement Questionnaire (PBQ) (Hunt & Padilla, 2006)

Q27 When answering the questions below, **think about the pet that you most recently rehomed.** (1 = Strongly Disagree, 5 = Strongly Agree)

I am very upset about my pet’s rehoming (1)

My life feels empty without my pet (2)

I feel lonely without my pet (3)

I miss my pet enormously (4)

I cry when I think about my pet (5)

I am very sad about rehoming my pet (6)

I’ll never get over the loss of my pet (7)

I feel angry towards animal professionals for not being able to help me keep my pet (8)

I am angry at my pet for making me rehome them (9)

I am angry at other people for contributing to the rehoming of my pet (10)

I am angry at my friends/family for not being more helpful (11)

I should have known that I couldn’t keep my pet (12)

I feel very guilty for not taking better care of my pet (13)

I feel bad that I didn’t do more to keep my pet (14)

I wish I had shown my pet more love (15)

I am dreaming about my pet a lot these days (16)

I have had nightmares about my pet’s rehoming (17)

I feel numb when I think about my pet (18)

I have scary memories of my pet’s rehoming (19)

Memories of my pet’s rehoming haunt me (20)

I am embarrassed to tell my family and friends that I rehomed my pet (21)

I am scared of people finding out that I rehomed my pet (22)

I try to hide that I needed to rehome my pet (23)

I am ashamed to think about having rehomed my pet (24)

My life is easier now that my pet was rehomed (25)

I feel less burdened now that my pet is rehomed (26)

I am relieved to know that my pet is in a new home (27)

Because my pet was rehomed, I feel a weight off my shoulders (28)

I regret rehoming my pet (29)

I wish I hadn’t rehomed my pet (30)

If I did it all over again, I would keep my pet (31)

I feel remorse when I remember that I rehomed my pet (32)

To show that you are paying attention, please select "Strongly Disagree" as your answer (33)

End of Block: Pet Bereavement Questionnaire (PBQ) (Hunt & Padilla, 2006)

Start of Block: Attitudes toward shelter/rehoming

Q47 **Think about the pet that you most recently rehomed.**

Q46 Did you have other pet(s) in your home when you rehomed your pet?

- Yes, I did have other pet(s) (1)
- No, I did not have other pet(s) (2)

| 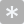 |
| --- |

Q28 Did you actively seek out a new pet after rehoming your pet?

- I did adopt/purchase a new pet after rehoming my pet (3)
- I did not adopt/purchase a new pet after rehoming my pet (10)

Display This Question:

If Did you actively seek out a new pet after rehoming your pet? = I did adopt/purchase a new pet after rehoming my pet

Q45 How long was it after you rehomed that pet until you purchased/adopted a new one?

- Less than 2 weeks (1)
- 2 - 4 weeks (2)
- 1 - 2 months (3)
- 2 - 6 months (4)
- 6 months - 1 year (5)
- 1 - 2 years (6)
- More than 2 years (7)

Q29 If you had to rehome another pet, which would be your most preferred method to rehome?

- Return the pet to the breeder (1)
- Rehome through a pet-specific website (e.g., Adopt a pet, Petfinder) (2)
- Rehome through personal networks (e.g., family, friends, acquaintances, co-workers) (3)
- Rehome through online advertisement (e.g., social media, Craigslist) (4)
- Rehome through a pet-specific rehoming website (e.g., Adopt a pet, Petfinder) (5)
- Bring the animal to a shelter or rescue (e.g., a physical facility) (7)
- Other (8) __________________________________________________

Q30 We would like to know your perspectives on animal shelters in general. Please rate how strongly you agree with the following statements: (1 = Strongly Disagree, 5 = Strongly Agree)

People should avoid relinquishing their pets to shelters or rescues (1)

I have seen a lot of negative stories in the news/online about animal shelters and rescues (2)

Shelter and rescue staff are knowledgeable about animal care (3)

Animal shelters and rescues help control pet overpopulation (4)

Animals are well cared for at animal shelters and rescues (5)

Animal shelters and rescues can assist me if I need help caring for my pet (6)

Animal control officers take pets away from good families (7)

The goal of animal shelters is to protect the public from nuisance animals and diseases that may jump from an animal to a human (e.g., rabies) (8)

The goal of animal shelters is to provide pets for adoption (9)

The goal of animal shelters is to protect animals from human cruelty and neglect (10)

End of Block: Attitudes toward shelter/rehoming

Start of Block: Demographic questions

Q31 What is your age? Write below in years.

________________________________________________________________

Q32 What is your gender identity?

- Woman (1)
- Man (2)
- Non-binary (3)
- Self-describe (4) __________________________________________________
- Prefer not to say (5)

Q33 What is your current employment status?

- Employed full time (40 or more hours per week) (1)
- Employed part time (up to 39 hours per week) (2)
- Unemployed and currently looking for work (3)
- Unemployed and not currently looking for work (4)
- Student (5)
- Retired (6)
- Homemaker (7)
- Self-employed (8)
- Unable to work (9)
- Other (10) __________________________________________________

Q34 Where do you live?

| Alberta (1) | Georgia (22) | New Mexico (43) |
| --- | --- | --- |
| Manitoba (2) | Hawaii (23) | New York (44) |
| British Columbia (3) | Idaho (24) | North Carolina (45) |
| Newfoundland and Labrador (4) | Illinois (25) | North Dakota (46) |
| Northwest Territories (5) | Indiana (26) | Ohio (47) |
| Nova Scotia (6) | Iowa (27) | Oklahoma (48) |
| Nunavut (7) | Kansas (28) | Oregon (49) |
| Ontario (8) | Kentucky (29) | Pennsylvania (50) |
| Prince Edward Island (9) | Louisiana (30) | Rhode Island (51) |
| Quebec (10) | Maine (31) | South Carolina (52) |
| Saskatchewan (11) | Maryland (32) | South Dakota (53) |
| Yukon (12) | Massachusetts (33) | Tennessee (54) |
| Alabama (13) | Michigan (34) | Texas (55) |
| Alaska (14) | Minnesota (35) | Utah (56) |
| Arizona (15) | Mississippi (36) | Vermont (57) |
| Arkansas (16) | Missouri (37) | Virginia (58) |
| California (17) | Montana (38) | Washington (59) |
| Colorado (18) | Nebraska (39) | West Virginia (60) |
| Connecticut (19) | Nevada (40) | Wisconsin (61) |
| Delaware (20) | New Hampshire (41) | Wyoming (62) |
| Florida (21) | New Jersey (42) |  |

Q35 Do you currently own pet(s)? If so, select all that apply

- Dog (domestic) (1)
- Cat (domestic) (2)
- Small animal (e.g., rat, guinea pig, rabbit, hamster, gerbil, mouse) (3)
- Exotic animal (e.g., bird, reptile, fish, amphibians, hybrids, arachnids, other wild species) (4)
- Large animal (e.g., horse, miniature pig, donkey, alpaca, llama, cow) (5)
- I do not currently own a pet (6)
- Other (7) __________________________________________________

End of Block: Demographic questions

Start of Block: Qualitative response

Q36 Please let us know if there’s anything else you’d like to tell us about your experience with rehoming a pet.

________________________________________________________________

End of Block: Qualitative response

Start of Block: End text

End text Thank you for participating in this survey! We appreciate your willingness to help. With this data, we will be able to improve the rehoming process for pets, owners, and adopters. Because there is limited research in the area of pet rehoming, there are no specific resources to help with the emotional impacts of rehoming your pet at this time. You might find the following resources about pet bereavement helpful to cope with the experience of pet rehoming.

 [Best Friends Animal Society - Pet Loss and Grief Resources Page](https://resources.bestfriends.org/article/pet-loss-and-grief-resources)

 [Ontario Veterinary College - Pet Loss Support Guide](https://pettrust.uoguelph.ca/petlossresources)

End of Block: End text
